# News and views on ion channels in cancer: is cancer a channelopathy?

**DOI:** 10.3389/fphar.2023.1258933

**Published:** 2023-11-14

**Authors:** Damian C. Bell, Luigi Leanza, Saverio Gentile, Daniel R. Sauter

**Affiliations:** ^1^ Sophion Bioscience A/S, Copenhagen, Denmark; ^2^ Department of Biology, University of Padua, Padua, Italy; ^3^ Department of Regenerative Medicine and Cell Biology, College of Medicine, Medical University of South Carolina, Charleston, IL, United States; ^4^ Sophion Bioscience Inc., Bedford, MA, United States

**Keywords:** ion channels, cancer, channelopathies, metastasis, apoptosis, patch-clamp technique, automated patch clamp (APC), electrophysiology

## Abstract

Ion channels are key signaling proteins found throughout the body; they are critical in many, wide-ranging physiological processes, from gene expression, sensory perception and processing to the cardiac action potential. When ion channel activity goes awry, for example, via mutation, damage or disrupted homeostasis, the outcome can result in causation, development and/or maintenance of disease. Ion channel dependent diseases have been dubbed channelopathies. Recent studies on the role of ion channels in cancer biology suggest that cancer is one such channelopathy. Many ion channels have now been implicated in the cellular processes that are affected in a multitude of cancers. In the last two decades, the field of ion channel and cancer research has been growing exponentially: a combination of developments in molecular biology, genetics, electrophysiology and automation have driven an explosion in our capabilities to interrogate ion channel pathways; how, why and where they go wrong and therapeutic interventions to correct their pathophysiology in cancer. A review of this vast and rapidly developing field would require a titanic tome to merely dimple the surface of research that has ballooned recently. In lieu of that huge undertaking—for the benefit of both authors and readers - this review discusses select examples of primary, applied and clinical research, aiming to shine a light on some of the more innovative and novel findings that this exciting field is excavating.

## Introduction

**FIGURE 1:**
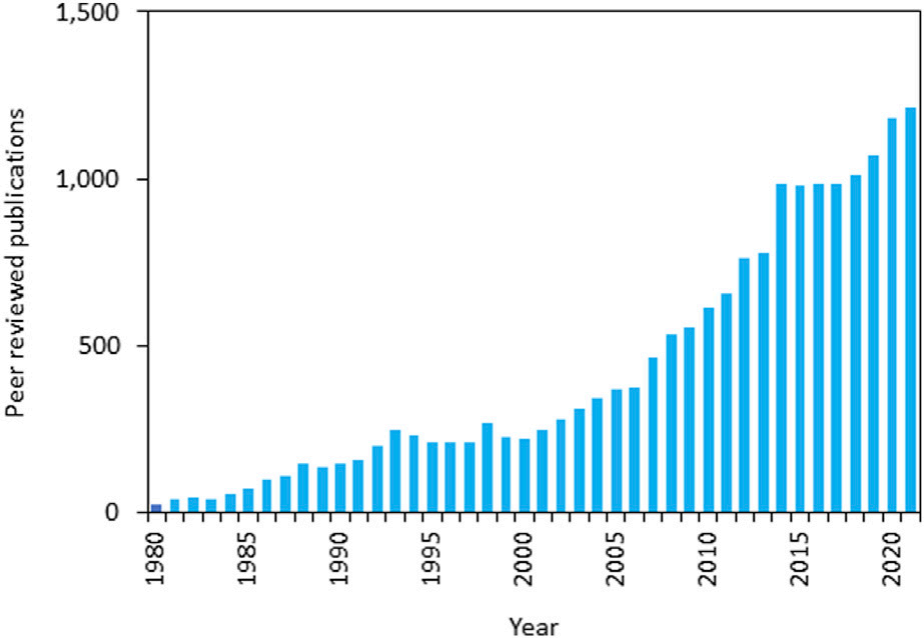
Forty years of ion channels and cancer publications - annual numbers of peer-reviewed publications from 1981 to 2021 (source: PubMed—https://pubmed.ncbi.nlm.nih.gov/; last accessed 19/07/2023 using search words “Ion channels” AND “Cancer”).

Ion channels and transporters are proteins involved in bioelectric properties of living cells and some of them are also able to modulate the intracellular ionic concentrations. The human genome includes at least 400 genes encoding for ion channels which are selectively expressed in the cellular compartments of all cells. Although ion channels have been traditionally studied for their role in controlling excitability in terminally differentiated cells such as neurons and cardiac myocytes, it is well understood that this class of proteins is involved in a large variety of biological processes ranging from cell proliferation, to metabolism and cell death. Alterations of biochemical signaling underlying these cellular processes are significant factors in carcinogenesis, thus ion channels are key factors in cancer biology. Nevertheless, research on the role of specific ion channels in controlling hallmarks of cancer has been sparse. Remarkably, in recent years, improvements of both conceptual framework and technologies have driven an exponential growth in the interest and understanding of roles of ion channels and cancer (see Figure 1).

**FIGURE 2:**
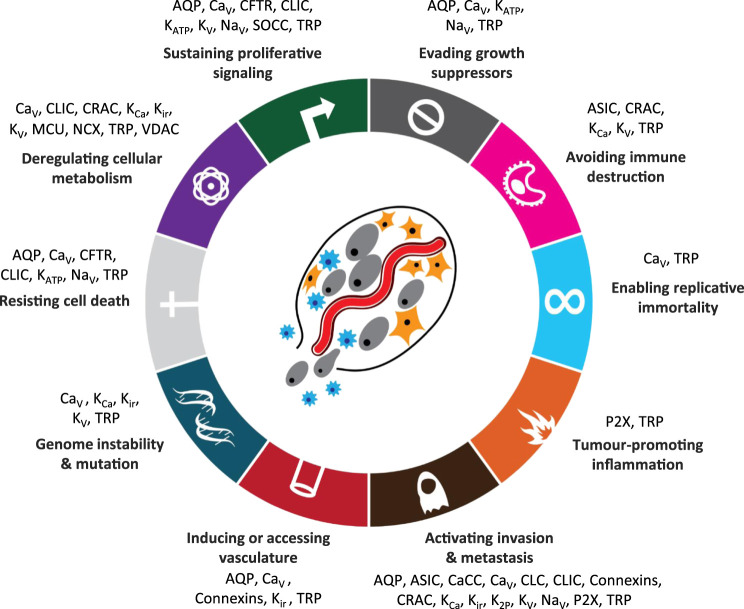
Hallmarks of cancer with associated ion channel families [ion channels added and adapted with permission from Hanahan (2022)]. Abbreviations: AQP, aquaporins; ASIC, acid sensitive ion channels; CaCC, calcium-activated chloride channels; CFTR, cystic fibrosis transmembrane conductance regulator; Ca_V_, voltage-gated calcium channels; CLC, chloride channels; CLIC, chloride intracellular ion channels; CRAC, calcium release-activated channels; K_ATP_, ATP-sensitive potassium channels; K_Ca_, calcium-activated potassium channels; K_ir_, inward rectifier potassium channels; K_2P_, two-pore domain potassium channels; K_V_, voltage-gated potassium channels; MCU, mitochondrial calcium uniporter; Na_V_, voltage-gated sodium channels; NCX, sodium-calcium exchanger; P2X, ATP-gated P2X receptors; SOCC, store-operated calcium channels; SUR, sulfonylurea receptor; TRP, transient receptor potential channels; VDAC, voltage-dependent anion channel.

**FIGURE 3:**
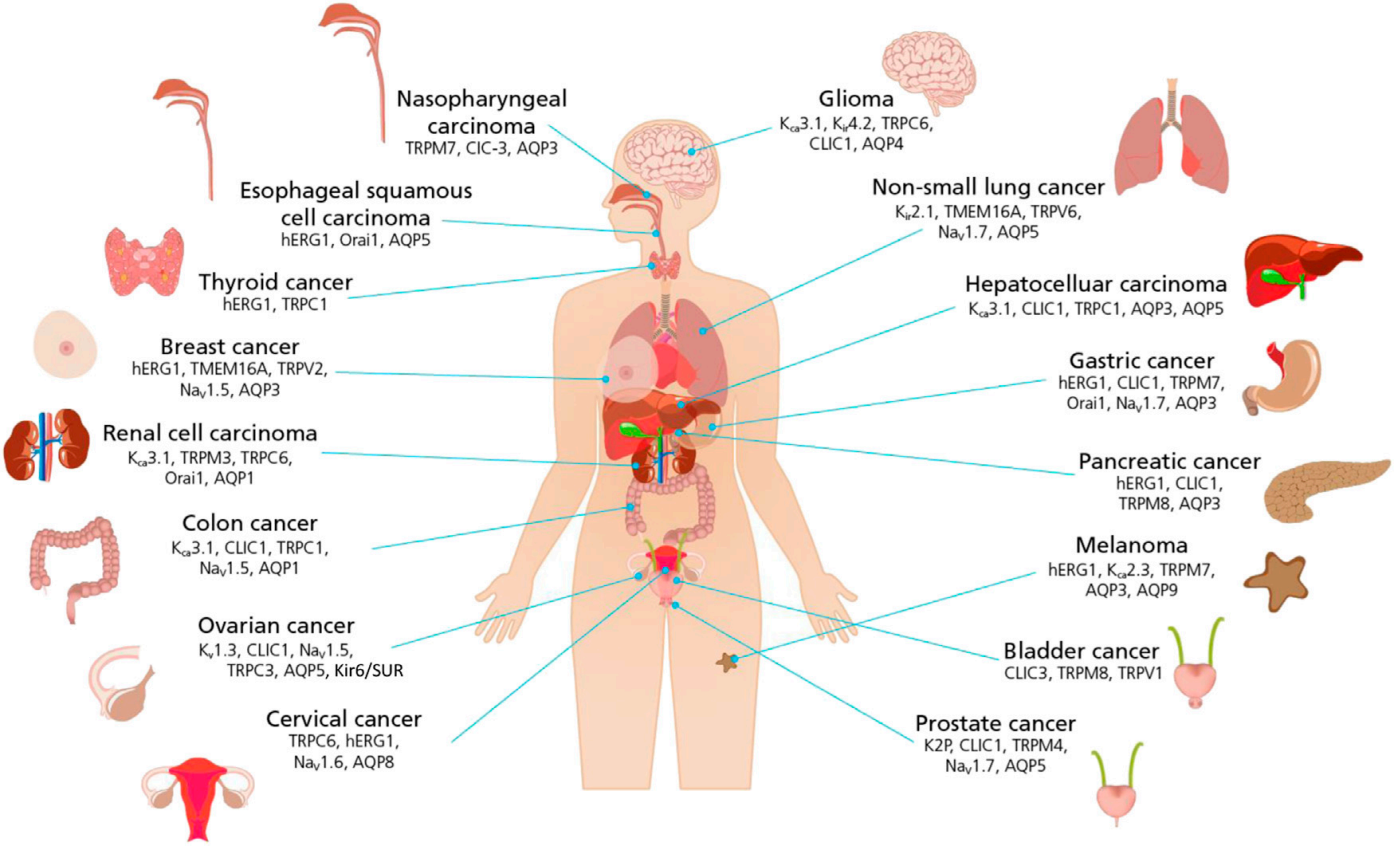
Schematic representation of the link between ion channels and cancers of different histogenesis. Cancer defined by body tissues and organs are pictured with a list of ion channels that have been found to play a significant role in controlling hallmarks of cancer. (For ion channel family abbreviations see Figure 2 legend).

For example, in the now seminal work by Hanahan and Weinberg (Hanahan and Weinberg, 2000; Hanahan and Weinberg, 2000) “hallmarks of cancer” were identified see Figure 2. Conceptually, each hallmark underlies distinct functional capabilities in promoting the carcinogenic processes ranging from sustaining proliferative signaling and evading death to cell metabolism. The past decade has seen the emergence of ion channels as key factors in controlling each of the hallmarks of cancer (Prevarskaya et al., 2018) and as a novel approach to cancer treatment. Defining cancers and their ion channels from another perspective: Figure 3 provides an overview of different cancers, alongside some of the key ion channels which expression and/or function alteration have been associated with carcinogenesis in each tissue or organ.

Using selected hallmarks of cancer, the current review aims to capture some of the innovative technologies, novel molecules and pathways that have been uncovered during the recent years, highlighting key advances in our understanding of ion channels, their role, involvement and identification as potential therapeutic targets in cancer. With such a vast vista of new findings to cover the authors have selected examples in each of primary, applied and clinical studies to highlight particular developments in the field.

## Primary research example 1—Wnt peptides in sustaining proliferative signaling and metastasis

Wingless-related integration site (Wnt) peptide ligands are the signaling molecules in Wnt pathways: signaling cascades shown to be critical in embryonic development, controlling processes like cell proliferation, differentiation and apoptosis (Azbazdar et al., 2021). When Wnt pathways become dysfunctional, loss of control in these key cellular processes can lead to several diseases, including cancers (Zhang and Wang, 2020).

Indeed, now for over three decades, Wnt peptides have been shown to be involved in cancer (for a review see Clevers and Nusse, 2012). Early studies made the link between Wnt signaling dysregulation and several hallmarks of cancer, including cell proliferation leading to uncontrolled tumor growth and metastasis. Remarkably, it has been shown that the biochemical cascades controlled by Wnt ligands including phosphoprotein cascades measured over minutes or hours (Klaus and Birchmeier, 2008) play a fundamental role in many of the distinct stages of the metastatic and/or cell proliferation processes (Li et al., 2018). More recently, accumulating data show a clear relationship between ion channels activity and Wnt signaling (Rapetti-Mauss et al., 2017; Strubberg et al., 2018; Breuer et al., 2019). Due to the nature of ion channels as “fast acting” proteins, studies in the role of ion channels in cancer offer the opportunity to investigate changes of cellular signaling on time scale of fraction of seconds. (Thrasivoulou et al., 2013). For example, the use of patch clamp techniques provides a resolution in the ms timescale that is needed to define the potential link between the initiation of signaling cascade events (e.g., Wnt peptide ligand binding to receptor) with the activity of a specific ion channel and vice-versa.

Thus, in a study by Ashmore et al. (2019), manual patch clamp (MPC) was used to study Wnt peptide binding and membrane potential changes at ms resolution. This study showed that, binding of the Wnt ligand to its surface membrane receptor of cancer cells produced a significant change of the membrane potential towards negative value (hyperpolarization) in a time window of about <100 ms (Ashmore et al., 2019). Interestingly, the authors found that Wnt-dependent hyperpolarization was achieved by the concerted activity of different ion channels that included activation of the BK potassium channel as downstream effector of changes of Ca^2+^ gradients (possibly upon opening of the TRPM8 Ca^2+^ channel).

**FIGURE 4:**
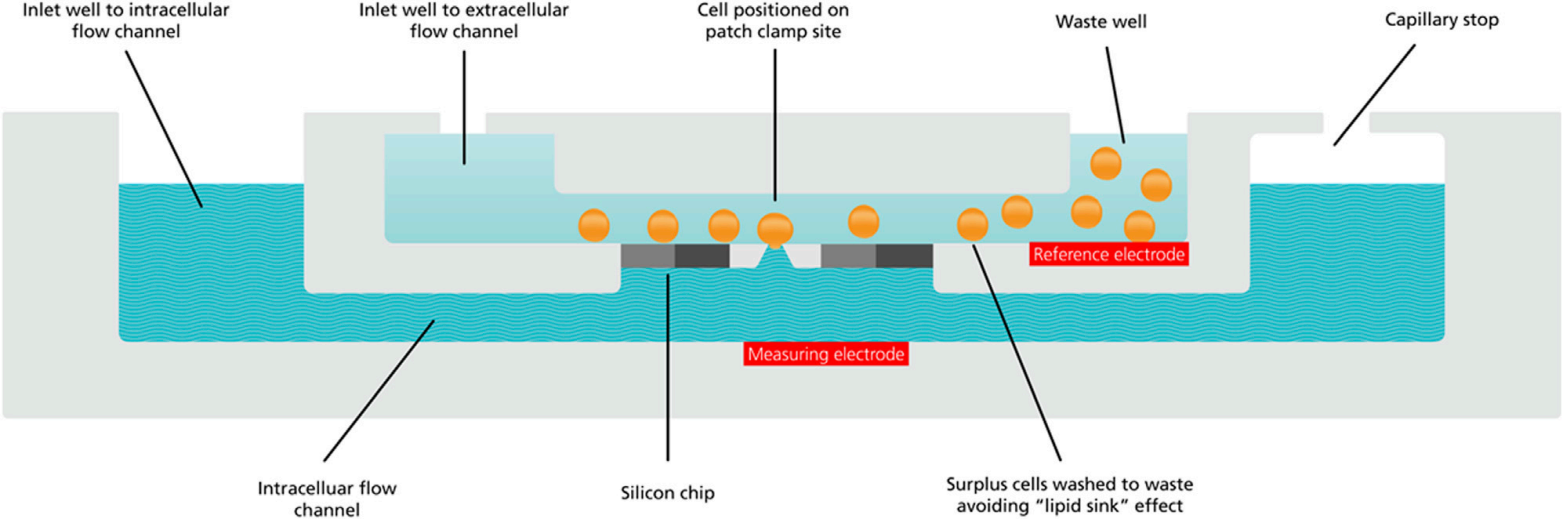
Cross section cut-away schematic of a QPlate recording well.

However, whilst MPC provided the temporal resolution to record the Wnt signaling cascade in few ms time scale-, this technique relies on the analysis of only one cell at the time in which the signaling can be investigated. Therefore, application of MPC requires a substantial investment in terms of time and technical skills that severely limit the numbers of recordings to make statistical analyses sufficiently powerful to tease apart subtle modulating activities. Therefore, to gain better understanding of the subtle and variable nature of these “fast events” and to give sufficient statistical power to accurately report Wnt peptide driven ion channel activity the authors duly turned to automated patch clamp (APC). Using this novel application of APC technology, the authors showed that even low concentrations (2 nM) of Wnt peptides had subtle, slow-developing ion channel activating effects that initiated the Wnt signaling cascade in cancer cell lines, effects they argued would likely be missed using MPC due to the low number of biological repeats that can be feasibly obtained with this approach (Ashmore et al., 2019). APC not only provided vastly increased recording throughput and statistical power (increased *n*), but also low volume, rapidly exchanged and steady-state applications of scarce Wnt peptide directly at the planar patch clamp recording site. Each recording site is fed by a microfluidic channel to apply low volumes (µLs) of recording solutions directly to the recording site micro-environment (see Figure 4).

The schematic shows the microfluidics inlets and throughflow waste reservoir for application of cell suspensions (orange spheres), intracellular (wavy blue pattern) and extracellular solutions (solid light blue) to the patch clamp recording site. In addition to the microfluidic flow channels, each recording well also houses the reference (labelled red rectangle) and measuring electrodes (labelled red rectangle), completing the electrical circuit between cell and feedback amplifier. This is one of 16 or 48 recording wells in a planar array in a recording plate (QPlate) used on the QPatch, an APC made by Sophion Bioscience.

## Primary research example 2—organellar Kv1.3 ion channels in cancer cell proliferation, apoptosis and tumorigenesis

Intracellular organelles—the endoplasmic reticulum (ER), Golgi apparatus, nucleus, endosomes, lysosomes and mitochondria - have several key cellular activities, like gene expression, cell metabolism, motility and apoptosis. Cancer cells often “hijack” such cellular processes, leading to dysfunctional metabolism, cell proliferation, apoptosis and ultimately tumorigenesis.

Several ion channels are found in organelles and their dysfunction can lead to disease, including metabolic and neurodegenerative diseases and cancers (Festa et al., 2022; Gururaja Rao et al., 2020; for reviews on organellar ion channels and cancer see: Leanza et al., 2013, Leanza et al., 2019, Peruzzo et al., 2016; Szabo et al., 2021). Table 1 summarizes the key organelles, ion channels and their roles in physiology and cancer.

**TABLE 1:** Key organellar ion channels and roles in cancer.

Organelle	Ion channels	Physiological roles	Cancer roles	References
ER/Golgi	IP3R, BK_Ca_, SK_Ca_, K_V_1.3, STIM1, TRPM8, CLIC1,2,4	Ca^2+^ homeostasis	Tumorigenesis, Apoptosis	Leanza et al. (2013); Peruzzo et al. (2016)
		Gene expression		
		Peptide post-translational modification		
Nucleus	IP3R, RyR, VDAC1, K_V_10.1, BK_Ca_, Kv1.3, CLIC1-5	Nuclear Ca^2+^ signaling	Tumorigenesis, Apoptosis	Leanza et al. (2013); Gururaja Rao et al. (2020)
		Transmembrane potential		
		Gene control and transcription		
		ATP transport		
Lysosome/endosome	TRPML1-3, TPC1-2, P2X4, K_V_10.1, BK_Ca_, TMEM175, CLIC1,4	Cell homeostasis—Ca^2+^ handling Degradation	Cell proliferation, Apoptosis	Gururaja Rao et al. (2020); Festa et al. (2022)
		Recycling		
		Autophagy		
		Cell death and proliferation		
		Cell defence and immunity		
Mitochondria	BK_Ca_, IK_Ca_, K_ATP_, Kv1.3, TASK3, MCU, VDAC1-3, CLIC1,4,5, PTP	Cell metabolism	Tumorigenesis, Cell proliferation, Apoptosis, Chemoresistance	Leanza et al. (2019); Gururaja Rao et al. (2020); Szabo et al. (2021)
		Ca^2+^ handling		
		Redox signaling		
		Cell death		

Our capabilities to record organelle ion channel (OIC) currents has made significant technological progress in recent years: unlike typical cell plasma membrane channel recordings, organelle ion channel recordings on membranes *inside the cell* require highly developed and adapted manual patch clamp (MPC) techniques (Chen et al., 2017; Shapovalov et al., 2017; Bednarczyk et al., 2021; Festa et al., 2022). Another area that has sprung from this foundational MPC research is the technological advancement in OIC recordings made via automated patch clamp (APC) platforms. There are two key methods emerging in APC recordings: overexpression of OICs in mammalian cells, using cell plasma membrane expression as a proxy for organellar membrane expression (e.g., TRPML1 expressed in HEK293T cells, Sauter et al., 2023); or, via selective purification and chemical manipulation of extracted organellar vesicle populations (Chen et al., 2017; Shapovalov et al., 2017) that can be used directly for OIC current recordings.

With these improved techniques numerous organellar ion channels have been identified as key elements in cellular dysregulation leading to cancers. Focusing on K_V_1.3 ion channels - found in the Golgi apparatus, nucleus and mitochondria - studies have shown the increasing importance of K_V_1.3 channel involvement in cancer tumorigenesis, cell proliferation and apoptosis (Prosdocimi et al., 2019; Teisseyre et al., 2019).

Recent research provides insights into the paradoxical, dual role of K_V_1.3. It has long been known that cell membrane located K_V_1.3 plays a key role in the *activation and proliferation* of immune cells (leukocytes and T cells). Conversely, mitochondrial K_V_1.3 has been shown to *promote cell death and apoptosis* in cancer cells (Szabó et al., 2008; Pérez-Verdaguer et al., 2015; Leanza et al., 2017). A key element in these processes of cell proliferation and cell death is caveolin1 (Cav1), a molecular determinant of the cellular location of K_V_1.3 ion channels. When K_V_1.3-Cav1 form a complex, the resulting K_V_1.3 lipid-rafts translocate K_V_1.3 to the cell plasma membrane. The resulting increase in channel density activates and promotes proliferation of leukocytes. When Cav1 is depleted, the cellular K_V_1.3 population swings towards greater density in the mitochondria: these mito-K_V_1.3 channels promote apoptosis (Capera et al., 2021). Thus, with such critical physiological and pathophysiological roles, K_V_1.3 ion channels are promising therapeutic targets (Varanita et al., 2022).

## Applied research example 1—Kv11.1 in cancer cell proliferation

The concerted activities of ion channels leading to variation of ionic gradients is a fundamental factor that controls a wide range of biochemical signaling in virtually all cellular processes. Remarkably, membrane potential of highly proliferative cells is more depolarized when compared with differentiated cells of the same type or terminally differentiated cells such as neurons or cardiac myocytes (Tokuoka and Morioka, 1957; Marino et al., 1994; Kadir et al., 2018). Furthermore, it has been established that each phase of the cell-division cycle is characterized by a specific membrane potential and progression from a given phase to the next is promoted by either depolarization or hyperpolarization. These events demonstrate that time-dependent activity of ion channels play a critical role in the cell-division cycle.

Based on these observations, the Gentile lab tested the hypothesis that imposing chronic hyperpolarization to cancer cells via pharmacological stimulation of potassium channels arrests proliferation in cancer cells. Interestingly, it has been found that stimulation of the Kv11.1 channel with activator molecules produced an arrest in the G0/G1 phase of the cell-division cycle (Lansu and Gentile, 2013). This event occurred in different cancer cells that are representative of breast (Lansu and Gentile, 2013), skin (Perez-Neut et al., 2016) and colon (Eskandari et al., 2021) cancer indicating that Kv11.1 hyperactivity affects cancer cell proliferation independently of their histogenesis. Furthermore, a large variety of proliferation markers and signaling were severely altered in these cells. For example, proteins promoting cell-cycle division (e.g., cyclins; oncogenes) were found significantly downregulated while tumor suppressors where upregulated (Lansu and Gentile, 2013; Perez-Neut et al., 2016; Breuer et al., 2019; Eskandari et al., 2021). These events were confirmed in *in vitro*, *in vivo* and *ex vivo* investigations including breast cancer samples directly derived from patients (patient-derived organoids; PDO) revealing that cancer cells/tumors exposed to Kv11.1 agonists activated a senescent-like phenotype without generating significant side effects. Although these cells are metabolically active, cellular senescence is characterized by a permanent loss of the proliferative ability thus it is considered a powerful anticancer mechanism (Schmitt et al., 2022). In conclusion, alteration of membrane potential by hyperpolarizing agents could be considered as a potent and safe anticancer strategy.

## Applied research example 2—Nav1.7 in activating invasion and metastasis

The voltage gated sodium channel (VGSC) Na_V_1.7 has long been studied in nociceptive (pain) signaling pathways [reviewed in Dib-Hajj et al. (2013)]. Unsurprisingly, with this prominent role in nociception, there have been several drug discovery and development programmes targeting Na_V_1.7 for non-opioid, non-addictive chronic pain medications (Chambers et al., 2016; Price et al., 2017; Rothenberg et al., 2019; Labau et al., 2020; Siebenga et al., 2020).

Building on this wealth of Na_V_1.7 primary and applied research, Pukkanasut et al. (2023) recently showed that Na_V_1.7 was selectively over-expressed in medullary thyroid cancer (MTC) but was not found in non-cancerous thyroid tissue. The group used both immortalized MTC cell lines and tissue from cancer patients to confirm this overexpression.

Researchers at the University of Alabama at Birmingham developed five small molecules that inhibit VGSCs in the micromolar range. Using these compounds, the group demonstrated that inhibition of the Na_v_1.5 in breast cancer cells (Dutta et al., 2018), resulted in a significant decrease in cell invasion. Pukkanasut et al. (2023) used one of these compounds, SV188, to assess its effect on MTC cell invasion and found that the compound inhibited invasion at a similar level as in the breast cancer cells. However, in MTC cells, Na_v_1.5 was only expressed at low levels. In contrast, prominent expression of Na_V_1.7 was detected in different immortalized MTC cell lines. This motivated the authors to test the effect of SV188 on Na_V_1.7 (Pukkanasut et al., 2023).

To record the isolated effect of the compound on Na_V_1.7, the group recorded currents in the whole cell patch clamp configuration from HEK293 cells recombinantly expressing Na_V_1.7. SV188 inhibited Na_V_1.7-mediated current with an IC_50_ of 3.6 µM, in good agreement with the cell viability assay in cancer cells. Patch clamp is a powerful technique that allows a detailed investigation of compound mechanism of action on the channel activity. Using specific voltage-pulse protocols, the group showed that SV188 is both a voltage- and use-dependent inhibitor of Na_V_1.7 that causes a hyperpolarization shift of the activation curve.

The study concluded that Na_V_1.7 might serve as a potential target to develop novel therapies for MTC. Furthermore, the authors discussed the potential role for Na_V_1.7 as biomarker.

## Clinical research example 1—Kir6/SUR in deregulating cellular metabolism and repurposing the activator Minoxidil to treat ovarian cancer

As our understanding of the importance of ion channels across all hallmarks and cellular processes in cancer cell homeostasis grows, exemplified in the primary and applied research studies discussed above, so does the need to translate this to therapeutic discoveries in the clinic. The next examples aim to highlight such clinical translation.

Conventional drug discovery programs routinely require 10–14 years from target identification to approval; during this time companies invest a staggering $1.3–2.8B (Wouters et al., 2020). Drug repurposing offers a cheaper, and often faster, route to get a drug on the market. In this process, approved compounds with well-defined safety and toxicology profiles are tested on novel targets and/or novel indications to develop a new treatment. The Gentile lab at the Medical University of South Carolina pursued such a drug repurposing campaign on the channel complex of inward rectifier potassium channel (Kir6) and sulfunylurea receptor type 2 (SUR2) to address the unmet need for treatment options of gynecologic cancers.

Kir6.1 or Kir6.2 are the functional units for which either SUR1 or SUR2 are necessary activating subunits. The selective expression of the sulphonylurea receptor (SUR) subunits makes the channel relevant for specific physiological functions. The Ki6/SUR1 complex is best known for its involvement in insulin secretion while Kir6/SUR2 activity plays a role in regulation of the vascular tone (Quayle et al., 1988; Seino and Miki, 2004; Ashcroft, 2005). For the latter, the channel has successfully been pursued as a drug target. The FDA-approved antihypertensive drug Minoxidil (Loniten ^®^) is an activator of Kir6/SUR2. By binding specifically to SUR2, Minoxidil causes hyperpolarization of smooth muscle cells resulting in vasodilation by reducing Ca^2+^ influx through voltage-gated Ca^2+^ channels (Meisheri and Cipkus, 1987). Later, Minoxidil was commercialized for arresting hair loss (Rogaine ^®^).

In addition to its well-established physiological functions, Kir6/SUR has also been implicated in cancer. Aberrant expression of Kir6/SUR has been reported in several types of cancers, including prostate, breast, pancreatic, and gynecologic cancers. A study by the Gentile lab and collaborators (Fukushiro-Lopes et al., 2020) revealed a relationship between channel expression and clinical outcome where ovarian cancer patients with elevated channel expression had an increased survival compared to a low expression group. This data suggested that high activity of Kir6.2/SUR2 produced an anticancer effect. The authors used a xenograft mouse model to provide further evidence for the relevance of targeting this channel in ovarian carcinogenesis. They found that exposing systemic application of Minoxidil resulted in an almost complete arrest of tumor growth without significant side effects. A closer investigation into the mode of action of the compound revealed that activation of Kir6.2/SUR2 provides the driving force for increased Ca^2+^ entry resulting in a deregulation of metabolism and increased mitochondria-dependent reactive oxygen species (ROS) production (Fukushiro-Lopes et al., 2020). An overload of ROS can trigger extended DNA damage and apoptosis underly the growth arrest in the xenograft model.

Although no targeted therapy has yet been universally accepted against ovarian cancer, several therapeutic approaches including PARP inhibitors and/or platinum-based chemotherapy provide initial benefits to ovarian cancer patients. However, the overall survival of these patients is still extremely limited as about 70% of ovarian cancer patients succumb to the disease within 5 years from diagnosis because of drug toxicity and eventually all patients develop drug resistance. Therefore, repurposing a Kir6/SUR2 activator drug with a safe pharmacological profile as Minoxidil can provide an unprecedented opportunity for the unmet need in treating ovarian cancer patients and other. Based in these premises, the Gentile lab and collaborators at the Loyola University Chicago and Medical University of South Carolina have recently activated a clinical trial to treat ovarian cancer patients with minoxidil.

Nevertheless, ABBC9 gene is a negative prognostic factor in breast and other cancers, and as such it is clear that novel selective drugs are needed (Maqoud et al., 2020, 2023). Furthermore, KCNJ11, KCNJ8, and ABCC9 genes are upregulated in different cancer types but ABCC8 is downregulated in pancreatic cancer (Maqoud et al., 2023). Also, sulfonylureas and glinides blocking the pancreatic Kir6.2-Sur1 subunits showed a higher risk for pancreatic cancer in line with the positive prognostic role of the ABCC8 gene but low risks for common cancers. Glibenclamide, repaglinide, and glimepiride show a lower cancer risk within the KATP channel blockers. An elevated expression of the Sur2A subunit was found in proliferating cells in two animal models of cancer; immunohistochemical reactivity to Sur2A-mAb was detected in the cytosol of the Ki67+/G3 cells other than in the surface membrane in the minoxidil-induced renal tumor and the canine breast tumor samples. In addition, pituitary macroadenoma has been observed in Cantú syndrome, which is associated with the gain-of-function mutations of the ABCC9 and KCNJ8 genes. Therefore, although these data support the notion that cancer can be considered channelopathies (Marques et al., 2018; McClenaghan and Nichols, 2022), the role of a specific ion channels in controlling oncosuppressor or carcinogenic signatures is cancer context dependent.

## Clinical research example 2—TRPV6 in calcium homeostasis, gene expression and sustained proliferative signaling—SOR-C13 clinical trial

TRPV6 are calcium selective ion channels. Over-activity or over-expression of TRPV6 leads to sustained, elevated levels of intracellular calcium ions; this loss of calcium homeostasis can have multiple effects in the cell including altered gene expression. One such calcium-dependent gene expression is the transcription factor Nuclear Factor of Activated T cell (NFAT). Elevated TRPV6 has been seen in several cancers (e.g., breast, ovarian, prostate, and pancreatic) and its activity is linked to the calcium-dependent transcription of NFAT which has been identified as a key pathway in these cancers (Haustrate et al., 2023; Lehen’kyi et al., 2012; Nilius and Szallasi, 2014; Tran Quang et al., 2015).

There is a vast and growing canon of animal and plant venom peptide toxins used as pharmacological tools and as potential therapeutics targeting ion channels (for reviews see: Cardoso, 2020; Dueñas-Cuellar et al., 2020; Groome, 2023; Luo et al., 2022; Mendes et al., 2023; Nogueira Souza et al., 2023; Osipov and Utkin, 2023; Talukdar et al., 2023; Wulff et al., 2019). Soricin, one such toxin from the Northern short-tailed shrew (*Blarina brevicauda*), shortened to the C-terminal 13mer peptide (SOR-C13), was shown to be a potent TRPV6 blocker (IC_50_ = 14 nM). Further, SOR-C13 binding and blocking of TRPV6 channels inhibited tumors in ovarian and breast cancer xenograft models (Bowen et al., 2013). Before entering clinical trials, SOR-C13 was also shown to be safe in animal toxicology studies.

With this primary and applied research foundation, SOR-C13 entered the clinic. “First in human” phase 1 trials were completed on patients with solid tumors of epithelial origin: it was shown to be safe and well tolerated, and also shown to have anti-tumor activity in pancreatic cancer (Fu et al., 2017). In 2019, based on these promising phase 1 trials, Soricimed Biopharma Inc. the company developing SOR-C13, has now extended these clinical trials into phase 1b trials at University of Texas MD Anderson Cancer Center (MDACC). The US FDA has also granted SOR-C13 orphan drug status to treat ovarian and pancreatic cancers.

## Clinical research example 3—voltage-gated sodium channels in activating invasion and metastasis—lidocaine in an operable breast cancer clinical trial

Surgical interventions remain one of the most effective forms of treatment in oncology. However, even after successful removal of the primary tumor, recurrence remains a risk. Ion channels have been explored as targets to lower this recurrence risk. One such approach that is undergoing evaluation in the clinic is described below.

VGSCs are a family of proteins that allow sodium ions to enter cells in response to changes in voltage. VGSCs are essential for many cell functions, including muscle contraction, nerve conduction and pain sensation (e.g., Na_V_1.7; see *Applied Research Example 2*). VGSCs have also been found to be overexpressed in several cancer cells, and their activity was linked to cell migration and invasion (Roger et al., 2003; Fraser et al., 2004; House et al., 2010). In invasive breast cancer cells, cells evade from the primary tumor by enzymatically degrading and translocating across the extracellular matrix (ECM). This mechanism permits their entry into the bloodstream and subsequent colonization of distant organs (such as the lungs), where they develop secondary tumors known as metastases. The degradation of the ECM by malignant cells relies upon the formation of specialized protrusions, referred to as invadopodia, which are notably enriched in filamentous actin (F-actin). Na_V_1.5 was found to be densely expressed in invadopodia where the channels co-localize with Na^+^-H^+^ exchanger type 1 (NHE1) and promote proton extrusion. This process aids acidic cysteine cathepsins (Cath), released by cancer cells, which in turn leads to ECM degradation (Brisson et al., 2011).

A team at the Tata Memorial Hospital (Mumbai, India) led by Dr. Rajendra A. Badwe recently evaluated the effect of inhibiting VGSCs during surgery in patients with operable breast cancer on the recurrence of cancer. The study aimed to repurpose the VGSC inhibitor, Lidocaine, that has been universally used as local anesthetic since its approval by the FDA in 1948. During the surgery, 0.5% lidocaine was injected peritumoral prior to excision. The phase 3 trial enrolled 1,600 patients and results were published in April 2023 (CTRI/2014/11/005228; Badwe et al., 2023).

Patients treated with lidocaine during surgical intervention showed a statistically significant prolongation in disease-free survival (DFS) and overall survival (OS). The relative risk reductions associated with this therapeutic intervention were 26% and 29% for DFS and OS, respectively, when compared to the control group. Whilst this benefit was moderate, the results are clinically meaningful and open the possibility for further developments in the future. An important aspect of the study is that the intervention provides a low-cost, easily implementable, one-time procedure that can be practiced in nearly all parts of the world. A further simplifying factor is that no subgroups were identified wherein the results are significantly different from those of the full study population, suggesting a general benefit to all patients with operable breast cancer.

## Summary

Ion channels have critical and multi-faceted roles in cellular signaling pathways in key processes like cell division and cell death; consequently, they are inextricably linked to cancer. The large and growing body of evidence defining ion channels as critical factors in all of the “hallmarks of cancer,” coupled with novel developments in ion channel techniques and technologies, has led to an explosion of research in the last decade (see Figures 1, 2).

With such vast vistas of research, even the most voracious and fervent students of cancer ion channel studies would not be able to cover the field adequately. This review - via select examples of primary, applied and clinical research - attempts to give the reader a scintilla of the field, by covering some of the more interesting and innovative studies that have been done in recent years.

Thus, the authors’ aim was to provide a guide to exemplar key developments in our understanding and the latest tools used to prosecute ion channel targets, pathways, causes and maintenance of cancer. The proof is in the pudding, namely, the drug discovery and development pipeline: the three examples discussed here (clinical trials using Minoxidil, Soricimed and lidocaine), alongside several other companies developing ion channel cancer therapeutics (e.g., Celex Oncology, Biosceptre, Sorrento Therapeutics, Wex Pharmaceuticals). The picture painted by all these extensive research endeavours is promising, and surely a life-saving ion channel cancer drug or therapy is on the horizon.
